# A single session of action observation therapy versus observing a natural landscape in adults with chronic neck pain – a randomized controlled trial

**DOI:** 10.1186/s12891-023-07070-w

**Published:** 2023-12-19

**Authors:** Tala Al Shrbaji, Mário Bou-Assaf, Rosa Andias, Anabela G. Silva

**Affiliations:** 1 School of Health Sciences, University of Aveiro, Campus Universitário de Santiago, Aveiro, 3800-193 Portugal; 2 CINTESIS.RISE@UA, University of Aveiro, Campus Universitário de Santiago, Aveiro, 3810-193 Portugal; 3 CINTESIS.RISE@UA, School of Health Sciences, University of Aveiro, Edifício 30, Agras do Crasto - Campus Universitário de Santiago, Aveiro, 3810-193 Portugal

**Keywords:** Action observation, Neck Pain, Movement representation, Motor imagery

## Abstract

**Background:**

Action observation (AO) has emerged as a potential neurorehabilitation therapy for patients with neck pain (NP), but evidence of its effectiveness is scarce. This study aims to assess the effect of a single session of AO when compared to observing a natural landscape on NP intensity, fear of movement, fear-avoidance beliefs, neck muscles’ strength, pressure pain threshold, and tactile acuity.

**Methods:**

Sixty participants with NP were randomly allocated to the AO group (n = 30) or control group (n = 30). Both groups watched an 11-minute video: the AO group watched a video of a person matched for age and sex performing neck exercises, while the control group watched a video of natural landscapes. Neck pain intensity, fear of movement, fear-avoidance beliefs, tactile acuity, pressure pain thresholds, and neck muscle strength were assessed both at baseline and post-intervention. General linear models of repeated measures (ANCOVA of two factors) were used to explore between-group differences at post-intervention.

**Results:**

There was a significant main effect of time for pain intensity (p = 0.02; η2p = 0.09; within-group mean change and 95% CI: AO=-1.44 (-2.28, -0.59); control=-1.90 (-2.74, -1.06), but no time versus group interaction (p = 0.46). A time versus group significant interaction was found for one out of the six measurement sites of two-point discrimination and the neck flexors strength (p < 0.05) favoring the control group. No other statistically significant differences were found for the remaining variables).

**Conclusions:**

Results suggest a similar acute benefit for both a single session of AO and observing natural landscapes for promoting hypoalgesia, but no impact on kinesiophobia, fear-avoidance beliefs, or pressure pain thresholds. Also, AO had no positive effect on two-point discrimination and muscle strength. Further research is needed, with longer interventions.

**Trial registration:**

Clinialtrials.gov (NCT05078489).

## Background

Neck pain (NP) was documented among the worldwide leading causes of years lived with disability as the fourth leading cause of disability-adjusted life years [1, 2]. Mean NP lifetime prevalence can be as high as 50%, year prevalence can reach 37%, month prevalence is around 25% and point prevalence is about 10% [3]. Despite the high impact and prevalence, evidence suggests that multimodal non-pharmacological interventions may be effective for pain and disability in the short term, but not in the long term, particularly for pain [4].

More recently, interventions targeting the central nervous system instead of the peripheral tissues, such as those requiring mental practice or observation of movements without the actual movement execution have shown promising results across different clinical conditions, including NP. Mental practice interventions include motor imagery, action observation (AO) training, and mirror therapy [5]. A systematic review showed these interventions to have favorable results on pain, range of motion, maximal quadriceps strength, and general health status in patients with total knee arthroplasty [6]. Also, an umbrella review with meta-analysis showed mental practice interventions to decrease pain intensity for chronic musculoskeletal pain [7], and another systematic review cautiously concluded that mental practice interventions decrease pain intensity and disability and improve joint position sense in patients with chronic non-specific NP [8]. This last systematic review, further reported that AO was more effective than motor imagery, suggesting that this was due to potentially higher neural recruitment associated with the visual feedback in the AO intervention, while motor imagery relies only on the individuals’ ability to imagine the movement [8]. However, these promising conclusions on the effects of AO for patients with NP were based on only 3 samples of patients (four studies) with sizes per group that varied between 10 and 15 participants [7, 9–11]. Thus, further research is needed on the effects of AO for individuals with NP. Furthermore, the effects of AO have been limited to a small set of variables, and its impact on aspects such as fear of movement, cortical reorganization, or strength have not been assessed for patients with NP, despite the promising results of mental practice for other body segments and clinical conditions [12, 13].

AO involves the observation and perception of human movement based on the mirror neuron system [14, 15]. Mirror neurons discharge during action execution and through the observation of similar actions performed by another person [14]. Observing the actions performed by other people causes the activation of motor regions involved in executing the same actions and increases the excitability of the corticospinal motor system, which might lead to a top-down central mechanism that induces hypoalgesia [16]. It might also induce plastic changes in the central nervous system similar to physical training with improved motor output [9].

This study aims to assess the immediate effect of a single session of observing neck movements (AO) on NP intensity (primary aim), fear of movement, fear-avoidance beliefs, neck muscles’ strength, pressure pain threshold, and tactile acuity when compared to observing a natural landscape in individuals with chronic idiopathic NP (secondary aim). We hypothesized that AO would have a larger effect on decreasing pain intensity than observing a natural landscape.

## Methods

### Study design, ethics approval, and registration

This study was a randomized controlled trial with two groups (the AO group and the control group). It was approved by the Ethics Committee of the University of Aveiro (21-CED/2020). All participants signed an informed consent before entering the study. Before the enrollment of the first participant, the study was registered at clinialtrials.gov (NCT05078489) on 14/10/2021.

### Procedures

#### Participant recruitment, eligibility criteria, and sample size

Participants were recruited from the general community through advertising on social media and direct invitation by the principal researcher. They entered the study if (i) reporting chronic idiopathic NP, defined as a recurrent or persistent pain that lasted for more than 3 months, with no trauma or etiology/diagnosis associated, which arises anywhere between the superior nuchal line and an imaginary transversal line through the first thoracic spinous process and lateral borders of the neck [17]; and (ii) aged between 18 and 65 years old. Participants were excluded if they presented any of the following conditions: cervical fracture or/and subluxation; pathology of malignant or visceral origin that causes neck pain; infectious diseases; cervical myelopathy; cervical surgery; osteoporosis; vestibular pathology; neurological disorder/deficits; rheumatic autoimmune diseases; history of cancer; severe cervical trauma (i.e. automobile accident that had affected the cervical area; whiplash); severe injury; visual and hearing dysfunction not corrected by eyeglasses/contact lenses or a hearing aid. Eligibility criteria were ascertained by self-report.

*A priori* sample size calculation was performed using the GPower software version 3.1.9.2, considering a medium effect size (0.25), an alpha of 5%, and 95% power when using a multivariate analysis of variance. It was estimated that 30 participants were required in each group.

#### Randomization and allocation concealment

The participants were randomly assigned to the AO group and the control group through a computerized random list generator (https://www.randomizer.org/). A research team member who was not involved in the assessment or the intervention was held accountable for randomizing and maintaining the list. Treatment allocation was concealed until the baseline intervention was performed when an opaque envelope with the information on group allocation was open.

#### Instruments and procedures

Participants were assessed for age, gender, number of years of formal education, weight, height, and pain duration at baseline. In addition, participants were assessed for pain intensity and frequency, disability, catastrophizing, fear of movement, fear-avoidance beliefs, tactile acuity, and pressure pain threshold, both at baseline and post-intervention as detailed below. The person who assessed participants also administered the intervention and, therefore, was not blind to the participant’s group allocation.

#### Pain intensity, duration, and frequency

Pain intensity at the moment was assessed using a 10-cm Visual Analogue Scale (VAS), anchored “no pain at all” and “worst pain imaginable” [18]. Mean changes of 2 points represent meaningful clinical changes [19].

NP duration and frequency were determined with closed questions (pain duration: “For how long have you had pain in the neck region?”; response options: i) between 3 and 6 months; ii) between 6 months to 1 year; iii) between 1 and 2 years; iv) between 2 and 4 years; and v) more than five years; Pain frequency: “How many times, in the past week, did you feel neck pain?”; response options: (i) never; (ii) rarely (once per week); (iii) occasionally (2 to 3 times per week); (iv) many times (more than 3 times per week); (v) always).

#### Neck disability index

Disability was assessed using the Neck Disability Index (NDI). This instrument was originally developed by Vernon and Mior [20] and consists of 10 items scored using a 6-point Likert scale. Each item defines the progressive levels of pain and limitation in activity, ranging from 0 (no pain or disability) to 5 (very painful or maximal limitation). The maximum total score is 50 points and can be interpreted as: 0 to 14 points = no disability; 5 to 14 points = mild disability; 15 to 24 points = moderate disability; 25–34 points = severe disability; and 34 to 50 points = complete disability [21]. The NDI European Portuguese version has good internal consistency (α Cronbach’s of 0.95) and high test-retest reliability (intraclass correlation coefficients (ICC) [2,1] = 0.91) [22]. The minimal detectable change (MDC) is 13 points, and the minimal important change (MIC) is 6 points [22].

#### Pain catastrophizing scale

Pain catastrophizing was assessed using the Portuguese version of the Pain Catastrophizing Scale (PCS). The PCS is a 13-item scale with three subscales and participants are instructed to recall past painful experiences and to indicate to what level they have experienced each on a 5-point Likert scale: 0 (not at all) to 4 (all the time) [23, 24]. The total score is the sum of the individual items and ranges from 0 to 52, and higher scores are indicative of higher catastrophic thinking. The PCS is a reliable and valid measure with a high internal consistency (α ≥ 0,87) [25].

#### Tampa scale of kinesiophobia

Fear of movement was assessed with the 13-item Portuguese version of the Tampa Scale for Kinesiophobia (TSK) [26]. Each item is scored using a 4-point Likert scale varying from 1 (strongly disagree) to 4 (strongly agree). The final score ranges between 13 and 52 points, and higher scores indicate higher levels of perceived fear [27]. Different scores indicate different levels of severity, interpreted as ‘subclinical’ (13-22), ‘mild’ (23–32), ‘moderate’ (33–42), and ‘severe’ (43–52). A change in severity levels can be interpreted as a clinically meaningful change [27]. The standard error of measurement (SEM) and the MDC of TSK-13 in patients with chronic pain was 2.42 and 6.71, respectively [28].

#### Fear-avoidance beliefs questionnaire

Fear-avoidance beliefs were assessed with the Portuguese version of the 16 items Fear-Avoidance Beliefs Questionnaire (FABQ), originally developed by Waddell et al. [29]. The FABQ is composed of two subscales: fear-avoidance and physical activity (FABQ-PA) (5 items) and fear-avoidance and work (FABQ-W) (11 items). Each item is answered using a Likert scale ranging from: 0 (completely disagree) to 6 (completely agree) [29], and the higher the score the higher the level of beliefs. The work subscale has a maximum score of 42 and the physical activity subscale has a maximum score of 24. The Portuguese version showed high reliability (K of Cohen = 0.80) and internal consistency (α Cronbach’s of 0.88 for FABQ-W and 0.77 for FABQ-PA) [30]. A study with patients with chronic low back pain found a MIC of 7 points for the FABQ-W and of 4 points for the FABQ-PA [31].

#### Tactile acuity

Tactile acuity was assessed with the two-point discrimination (TPD) test using an aesthesiometer (Baseline Two-Point Aesthesiometer). This test assesses the ability of the individual to precisely perceive the location and quality of touch, which is assumed as an indirect measure of cortical reorganization [32]. The protocol used to assess TPD was adapted from two previous studies [33, 34]. Two-point discrimination measurements were taken centrally at the neck from six sites located between the third cervical vertebra and the first thoracic vertebra (C3-C4, C4-C5, C5-C6, C6-C7, C7-C8, and C8-T1). Three measures were taken at each level, after a familiarization trial in the arm, and the mean was used for statistical purposes. Participants were in the prone position while a mechanical caliper was applied at each point until the first blanching of the skin appeared around the tips of the caliper. The test started with 0 mm distance between the two tips of the caliper, gradually incrementing the distance by 1 mm until the participants recognized two points instead of one [35]. Participants were instructed to say ‘one’, when one point was felt, or ‘two’, when two points were felt, after every application [34]. Two-point discrimination at the cervical region demonstrated excellent test-retest reliability (ICC = 0.85) and a SEM of 3.7 mm [35].

#### Neck muscle strength

Neck muscle strength was assessed with a hand-held dynamometer (Advanced Force Gauge, 2500 N; Mecmesin, West Sussex, UK). Participants were briefed about the procedure and went through a trial in the limbs for familiarization. Before the neck muscles assessment, participants performed warm-up exercises by doing 10 active repetitions of neck flexion, extension, and right and left side flexion. The testing procedure consisted of three consecutive isometric contractions held for three seconds for each of the four neck movements (extension, flexion, right and left side flexion), with 30 s of rest between repetitions. For neck flexors, the head was held slightly above the plinth and with craniocervical flexion and participants were instructed to push against the dynamometer placed on the center of the forehead above the eyebrows [36, 37]. For neck extensors, participants were asked to assume a prone position with the shoulders placed at the edge of the plinth and the head held against gravity [37]. Participants were asked to hold their heads in a neutral position while pushing against the dynamometer positioned superiorly to the external occipital protuberance. For side flexors, participants were in the supine position and were instructed to maintain a neutral head position with the back of the head resting on the plinth, while pushing against the dynamometer centered on the ipsilateral side of the head [37].

Resistance was set at a rate of about 3 kg/s and the maximum isometric strength value was recorded at the peak of the participant’s ability to maintain contraction [36, 37]. Three measurements were taken of all tests and the mean was used for analysis. The hand-held dynamometer was found to have very good test-retest reliability (ICC between 0.94 and 0.97) and inter-session reliability (ICC between 0.87 and 0.95) [38]. Shahidi et al. [37] determined the MDC for the four neck muscle strength tests: neck flexors 8.7 (kgf), neck extensors 12.5 (kgf), right side flexors 7.2 (kgf) and left side flexors 6.3 (kgf).

#### Pressure pain threshold

Pressure pain threshold (PPT) was measured with an electronic pressure algometer (JTECH Medical Industries, Salt Lake City, US) at the right and left upper trapezius muscles (at the middle distance between the posterior angle of the acromion and C7), at the right and left articular pillar between C1 and C2 (1 cm laterally) and C5/C6 using previously published procedures [39]. A test trial run was conducted on the hypothenar region of the hand to familiarize participants with the procedure. Then, participants were positioned prone with their heads aligned and relaxed. The algometer was applied perpendicularly to each point and strength was applied at a rate of 3 N/s up to a maximum of 60 N. Measurements at each point were taken three times with 30–40 s rest in between. Participants were instructed to say the word “stop” as soon as the pressure sensation changed to pain. Pain pressure threshold algometry has demonstrated high intratester (ICC = 0.94–0.97) and interrater reliability (ICC = 0.79–0.90) [40]. A variation of 4.41 N is considered the MDC for PPT for the acute neck pain population [40].

#### Training of the assessors

Prior to the study, the assessors underwent a three-stage training procedure: (i) a three-hour session of training with a senior researcher, (ii) data collection in at least three participants who did not enter the final sample size, and (iii) a final session to clarify any doubts and difficulties from implementing the study protocol.

### Intervention and control

**Fig. 1:**
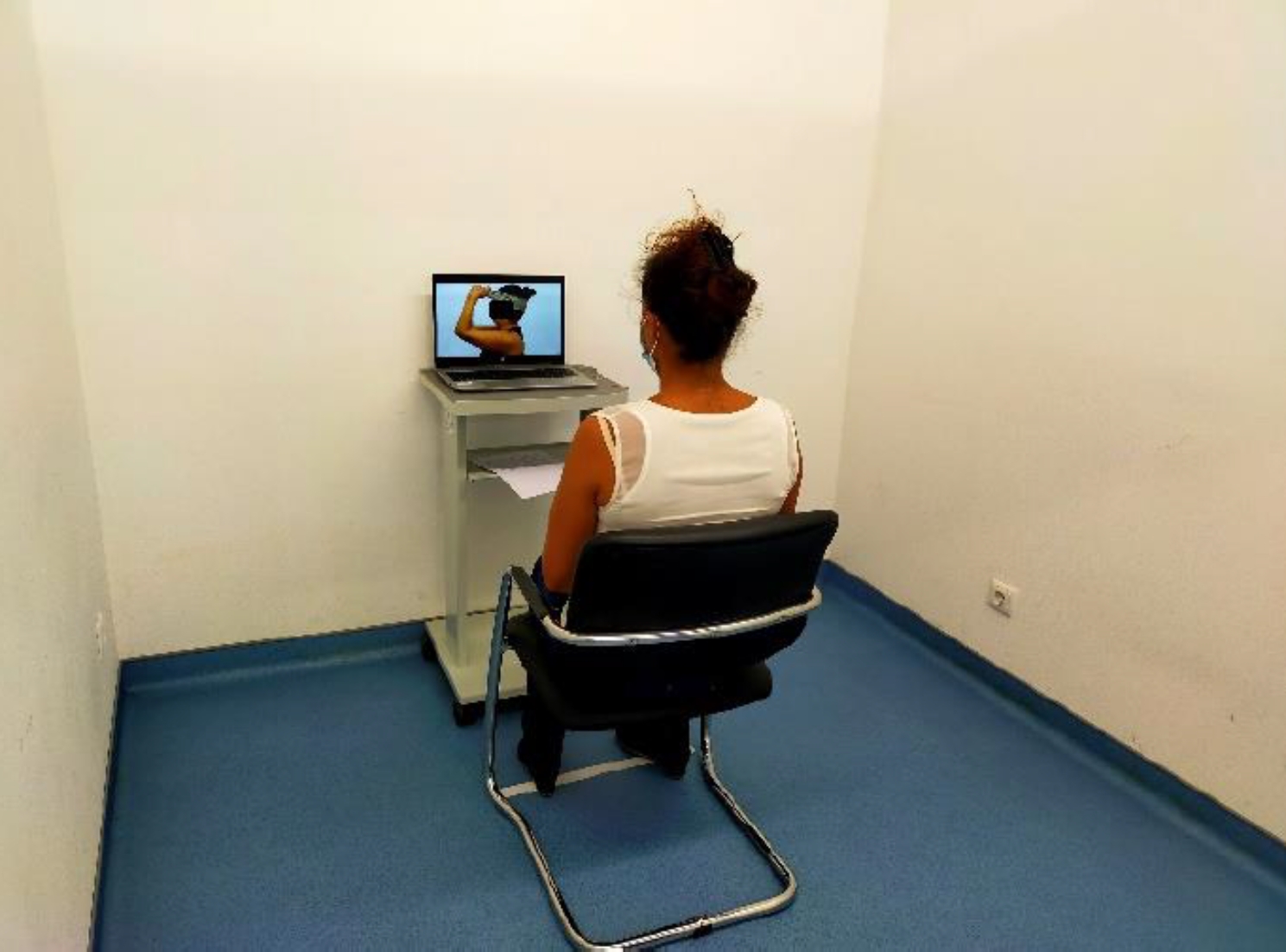
Setting of the intervention

Participants in both groups were instructed to sit on a comfortable chair in a quiet, empty room with white walls in front of a laptop (Fig. 1). They were asked to observe the respective video: the AO group observed a video of a person performing specific therapeutic neck exercises, and the control group watched a video of natural landscapes.

#### Action observation

**Fig. 2:**
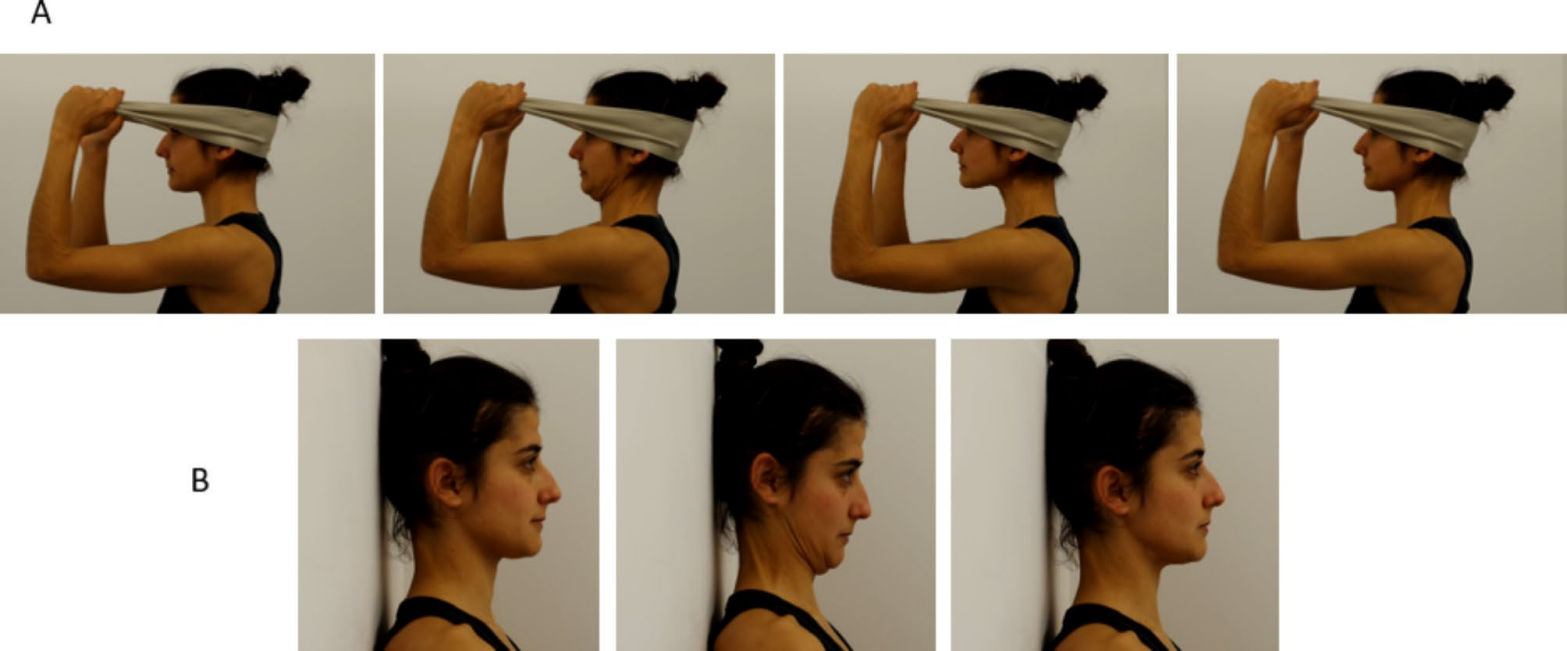
Neck exercises. **A**) Cranio-cervical flexion-extension exercise against resistance. **B**) Cranio-cervical flexion exercise

Participants were asked to observe video clips of a person performing two different exercises of craniocervical flexion (Fig. 2) with full attention and concentration without performing any movement during the observation time. Different versions of the video were produced for males and females and three age groups (18 to 33 years old, 34 to 48 years old, and 49 to 65 years old) and each participant was shown a video of a person from the same sex and age group. Age groups were defined based on Geifman et al. [41]. Exercises involved cranio-cervical flexion-extension movements with resistance and cranio-cervical flexion against a wall as reported in a previous study [10]. The video had a total of 11 min, divided as follows: (i) four minutes of observing clips of the exercises (two minutes for each exercise) performed by an individual of the same sex and similar age, (ii) followed by three minutes of rest, which consisted of a completely black screen so that participants would not be interrupted in this period, and (iii) repetition of the 4 min of the video clip of the exercise. This protocol was adapted from a previous study [10].

#### Control

**Fig. 3:**
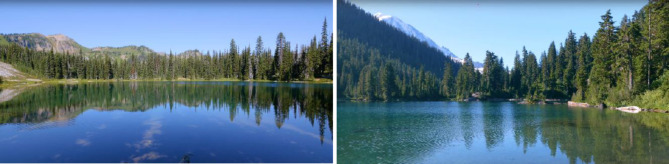
Natural landscapes

Participants in this group observed a video that displayed natural scenes with no human movement stimuli, similar to other previous studies [10, 42]. The protocol of the video for this group was similar to the AO group. The first four minutes were of a natural landscape with a lake (Fig. 3), then a three-minute interval with a completely black screen, and another four minutes of a video of a different natural landscape. The natural landscape videos were not static but rather moved from right to left with the presence of a light breeze onto the trees and moving water.

### Statistical analysis

All data analyses were performed using Statistical Package for Social Science (SPSS) 25.0 for Windows (SPSS Inc, Chicago, IL). Mean and standard deviation (SD) and count and proportion were used to describe continuous and categorical variables, respectively. Data were assessed for outliers, normality using Kolmogorov-Smirnov test and homogeneity of variance. Between-group differences for baseline characteristics were explored using a Student t-test (continuous variables) or a non-parametric equivalent (e.g.Mann-Whitney test), and using a Chi-square for nominal variables. A general linear model of repeated measures (ANCOVA of two factors) was used to assess the impact of the intervention on the outcome measures (i.e. VAS, TSK, FABQ-PA, FABQ-W, tactile acuity, and muscle strength). The within-subject factor was time (baseline vs. post-intervention), the between-subject factor was the intervention (AO vs. control), and covariates were sex, age, and baseline values of FABQ-PA. Partial eta squared was used as an indicator of effect size and interpreted as small (0.01), medium (0.0.06), and large (0.14) effect size [43]. The significance for all statistics was set at p < 0.05.

## Results

**Fig. 4:**
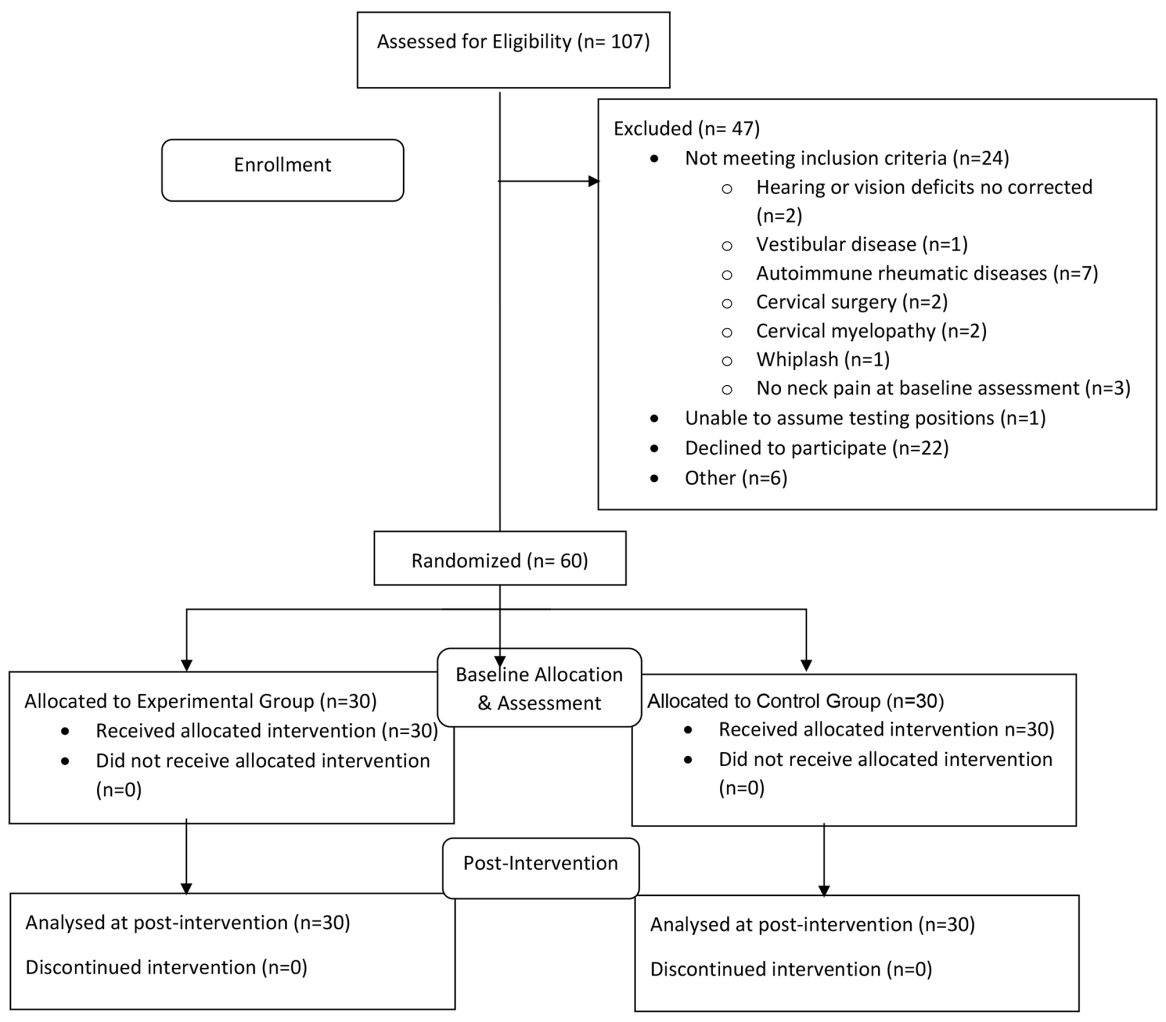
Flowchart of study participants

Of 107 individuals screened for eligibility, 24 were excluded because they did not meet the inclusion criteria, one was unable to assume the testing positions, 22 declined to participate and 60 entered the study (30 individuals in each group). The flow of participants is represented in Fig. 4.

### Sample characteristics

The AO group consisted of 24 females (80%) and 6 (20%) males, and the control group had 22 (73.3%) females and 8 (26.7%) males. The mean age (± SD) in the AO group was 37.4 (± 9.9) years old, and in the control group was 42.3 (± 10.2) years old (see Table 1).

**Table 1:** Sociodemographic characteristics

Variables	Control group (n = 30)	AO group (n = 30)	p-value
Sex (n, %)			
Female	22 (73.3%)	24 (80.0%)	0.761
Male	8 (26.7%)	6 (20.0%)	
Age (years; mean ± SD)	42.3 (± 10.2)	37.4 (± 9.9)	0.063
Weight (Kg; mean ± SD)	69.3 (± 12.8)	68.7 (± 11.4)	0.856
Height (cm, mean ± SD)	164.6 (± 9.3)	167.1 (± 8.3)	0.284†
BMI (Mean ± SD)	25.5 (± 3.9)	24.7 (± 3.9)	0.391
Education Level (n, %)			
Primary Education	2 (6.6%)	1 (3.3%)	0.817
Secondary Education	11 (36.7%)	14 (46.8%)	
Undergraduate Education Master’s degree Doctor Degree	11 (36.7%) 3 (10.0%) 3 (10.0%)	10 (33.3%) 4 (13.3%) 1 (3.3%)	

† Without normal distribution (non-parametric test corroborated the parametric test)

Abbreviations: AO- Action Observation; Kg- Kilograms; cm-centimeters; SD- Standard Deviation

A detailed characterization of the baseline clinical data of the sample is presented in Table 2. No between-group differences were found at baseline (p ≥ 0.05), except for fear-avoidance beliefs with individuals in the control group reporting significantly higher fear-avoidance beliefs related to physical activity than individuals in the AO group (p = 0.026).

**Table 2:** Participants’ characteristics at baseline

Variables		Control group (n = 30)	AO group (n = 30)	p-value
VAS (0–10), Mean (± SD)		5.08 (± 1.84)	4.88 (± 1.86)	0.682
Pain Frequency (n, %)	Rarely (1xweek)	3 (10.0%)	1 (3.3%)	0.125
	Occasionally (2/3xWeek)	8 (26.7%)	10 (33.3%)	
	Many times (> 3xWeek)	16 (53.3%)	11 (36.7%)	
	Always	2 (6.7%)	8 (26.7%)	
	Other	1 (3.3%)	0 (0.0%)	
Pain Duration (n, %)	Between 3 to 6 months	3 (10.0%)	2 (6.7%)	0.109
	Between 6 months to 1 year	3 (10.0%)	5 (16.6%)	
	Between 1 year to 2 years	3 (10.0%)	6 (20.0%)	
	Between 2 years and 5 years	10 (33.3%)	2 (6.7%)	
	More than 5 years	11 (36.7%)	15 (50.0%)	
NDI (0–50), Mean (± SD)		12.40 (± 4.28)	11.00 (± 4.76)	0.236†
PCS (0–52), Mean (± SD)		17.70 (± 11.10)	14.97 (± 10.88)	0.339
TSK (13–52), Mean (± SD)		28.20 (± 5.32)	26.80 (± 6.66)	0.372†
FABQ-PA (0–24), Mean (± SD)		9.50 (± 6.05)	6.17(± 5.21)	0.026
FABQ-W (0–42), Mean (± SD)		15.57(± 6.39)	16.13 (± 7.64)	0.756
TPD (cm), Mean (± SD)	C3 – C4	1.99 (± 1.33)	1.54 (± 1.41)	0.208†
	C4 – C5	1.82 (± 1.20)	1.73 (± 1.42)	0.795†
	C5 – C6	2.06 (± 1.44)	1.82 (± 1.58)	0.548†
	C6 – C7	2.27 (± 1.56)	1.86 (± 1.65)	0.327†
	C7 – T1	2.79 (± 1.95)	2.22 (± 1.66)	0.230†
	T1 – T2	2.85 (± 1.98)	2.37 (± 1.84)	0.337
PPT (N), Mean (± SD)	C1-C2 (R)	11.00 (± 6.42)	10.85 (± 5.71)	0.922†
	C1-C2 (L)	10.77 (± 5.66)	10.38 (± 4.52)	0.769
	C5-C6 (R)	11.21 (± 6.01)	11.50 (± 6.24)	0.853†
	C5-C6 (L)	10.47 (± 5.77)	11.04 (± 5.93)	0.705†
	Trapezius (R)	11.50 (± 5.11)	11.80 (± 5.69)	0.831†
	Trapezius (L)	10.83 (± 5.38)	11.06 (± 3.86)	0.848
Neck muscle strength (N), Mean (± SD)	Flexion	60.86 (± 22.32)	58.85 (± 16.77)	0.695
	Right Lateral Inclination	59.68 (± 21.17)	58.64 (± 14.32)	0.825
	Left Lateral Inclination	59.05 (± 21.47)	55.75 (± 13.86)	0.483
	Extension	98.32 (± 33.24)	95.31 (± 24.29)	0.690

† Without normal distribution (non-parametric test corroborated the parametric test)

Abbreviations: AO- Action Observation; FABQ-PA – Fear Avoidance Belief Questionnaire – Physical Activity Subscale; FABQ-W – Fear Avoidance Belief Questionnaire – Work Subscale; L- Left; NDI – Neck Disability Index; N – Newtons; PCS – Pain Catastrophizing Scale; PPT- Pressure Pain Thresholds; R- Right; SD- Standard Deviation; TPD - Two-point discrimination; TSK - Tampa Scale of Kinesiophobia; VAS- Visual Analogue Scale

### Post-intervention assessment

#### Neck pain intensity

There was a significant decrease in pain intensity from baseline to post-intervention (F [1, 53] = 5.51; p = 0.02; η2p = 0.09), but no interaction between time and group (F [1, 53] = 0.57; p = 0.46; η2p = 0.01). Table 3 shows the adjusted within-group mean differences.

**Table 3:** Adjusted within-group differences for pain intensity, fear of movement, fear-avoidance beliefs, tactile acuity, pressure pain thresholds, and muscle strength at baseline and post-intervention

Variables		Control group (n = 30)			AO group (n = 30)			Statistical results (p-values)					
		Pre-Treatment	Post-Treatment	Mean Difference & 95% CI*	Pre-Treatment	Post-Treatment	Mean Difference & 95% CI	Time	Group	Group*Time	Sex	Age	FABQ-PA
VAS (0–10)		4.96 [4.26, 5.65]	3.06 [2.10,4.02]	-1.90 [-2.74, -1.06]	5.01 [4.31, 5.70]	3.57 [2.61, 4.53]	-1.44 [-2.28, -0.59]	**0.02**	0.60	0.46	0.02	0.78	0.52
TSK (13–52)		27.27 [25.15, 29.39]	24.73 [22.28, 27.19]	− 2.54 [-3.91, -1.17]	27.73 [25.61, 29.85]	27.24 [24.78, 29.69]	-0.49 [-1.86, 0.87]	0.18	0.36	0.05	0.69	0.14	0.70
FABQ-PA (0–24)		9.42 [7.33, 11.52]	9.33 [7.11, 11.52]	0.09 [-1.66, 1.48]	6.24 [4.15, 8.33]	7.09 [4.88, 9.29]	+ 0.85 [-0.72,2.37]	0.40	0.07	0.39	0.23	0.67	
FABQ-W (0–42)		14.75 [12.13, 17.38]	12.64 [9.89, 15.39]	− 2.11 [-3.43, -0.80]	16.95 [14.32, 19.57]	15.13 [12.38, 17.88]	− 1.82 [-3.13, -0.50]	0.61	0.23	0.76	0.11	0.50	0.70
TPD (cm), Mean (± SD)	C3 – C4	1.77 [1.29, 2.25]	1.31 [0.84, 1.79]	-0.46 [-0.74, -0.17]	1.77 [1.29, 2.24]	1.83 [1.36, 2.30]	+ 0.06 [-0.22,0.34]	0.30	0.45	**0.02**	0.37	0.75	0.88
	C4 – C5	1.59 [1.14, 2.04]	1.40 [0.93, 1.87]	-0.19 [-0.49, 0.12]	1.97 [1.52, 2.42]	1.70 [1.23, 2.18]	-0.27 [-0.58, 0.04]	0.96	0.29	0.72	0.85	0.95	0.13
	C5 – C6	1.80 [1.29, 2.32]	1.45 [0.94, 1.96]	-0.35 [-0.59, -0.12]	2.07 [1.56, 2.59]	1.93 [1.41, 2.44]	-0.14 [-0.38,0.09]	0.22	0.31	0.22	0.44	0.74	0.99
	C6 – C7	2.00 [1.46, 2.54]	1.62 [1.06, 2.18]	-0.38 [-0.74, -0.02]	2.12 [1.58, 2.66]	2.02 [1.46, 2.58]	− 0.10 [-0.46,0.26]	0.97	0.49	0.29	0.86	0.99	0.38
	C7 – T1	2.54 [1.91, 3.18]	2.13 [1.46, 2.80]	-0.41 [-0.82, -0.01]	2.47 [1.84, 3.11]	2.38 [1.71, 3.04]	-0.09 [-0.50,0.31]	0.49	0.85	0.29	0.40	0.67	0.40
	T1 – T2	2.58 [1.91, 3.26]	2.36 [1.67, 3.05]	-0.22 [-0.63, 0.18]	2.64 [1.97, 3.31]	2.63 [1.94, 3.32]	-0.01 [-0.42,0.39]	0.66	0.74	0.49	0.92	0.62	0.62
PPT (N), Mean (± SD)	C1-C2 (R)	11.00 [8.64, 13.36]	11.16 [8.64, 13.68]	+ 0.16 [-0.94, 1.26]	10.85 [8.49, 13.21]	10.00 [7.48, 12.52]	-0.85 [-1.95, 0.25]	0.55	0.71	0.22	0.19	0.87	0.77
	C1-C2 (L)	10.79 [8.81, 12.77]	10.64 [8.39, 12.89]	-0.15 [-1.16, 0.84]	10.36 [8.38, 12.34]	10.03 [7.78, 12.28]	-0.33 [-1.33, 0.67]	0.48	0.73	0.81	0.32	0.53	0.54
	C5-C6 (R)	11.24 [8.89, 13.59]	11.04 [8.71, 13.37]	-0.20 [-1.18,0.78]	11.46 [9.11, 13.81]	11.32 [8.98, 13.65]	-0.14 [-1.13,0.83]	0.53	0.88	0.94	0.42	0.42	0.14
	C5-C6 (L)	10.47 [8.19, 12.75]	10.57 [8.38, 12.76]	+ 0.10 [-0.87,1.08]	11.04 [8.77, 13.32]	11.13 [8.94, 13.32]	+ 0.09 [-0.89,1.06]	0.91	0.73	0.98	0.19	0.38	0.36
	Trapezius (R)	11.53 [9.45, 13.61]	11.14 [9.03, 13.26]	-0.39 [-1.59,0.82]	11.78 [9.70, 13.86]	11.34 [9.23, 13.46]	-0.44 [-1.64,0.77]	0.64	0.88	0.96	0.32	0.35	0.56
	Trapezius (L)	11.22 [9.46, 12.98]	11.73 [9.74, 13.72]	+ 0.51 [-0.44, 1.46]	10.68 [8.91, 12.44]	10.75 [8.76, 12.74]	-0.07 [-0.88,1.02]	0.43	0.57	0.53	0.02	0.76	0.11
Neck muscle strength (N), Mean (± SD)	Flexion	62.12 [55.31, 68.93]	64.71 [57.51, 71.91]	+ 2.59 [-0.28,5.45]	57.59 [50.78, 64.40]	55.57 [48.37, 62.77]	-2.02 [-4.89,0.84]	0.86	0.18	0.03	0.96	0.81	0.30
	Right Lateral Inclination	60.38 [53.64, 67.12]	62.71 [55.88, 69.54]	+ 2.33 [-0.79,5.45]	57.93 [51.19, 64.67]	55.83 [49.00, 62.66]	-2.10 [-5.22,1.02]	0.43	0.34	0.06	0.03	0.40	0.52
	Left Lateral Inclination	59.72 [52.86, 66.57]	61.39 [53.99, 68.79]	+ 1.67 [-1.36,4.71]	55.08 [48.23, 61.94]	54.57 [47.17, 61.98]	-0.51 [-3.55,2.52]	0.23	0.27	0.33	0.02	0.69	0.54
	Extension	100.33 [89.52,111.14]	100.25 [88.69,111.82]	-0.08 [-4.11,3.96]	93.30 [82.49, 104.11]	88.88 [77.31,100.44]	-4.42 [-8.46,-0.40]	0.31	0.26	0.15	0.46	0.06	0.26

Abbreviations: AO- Action Observation; CI -Confidence Interval; FABQ-PA – Fear Avoidance Belief Questionnaire – Physical Activity Subscale; FABQ-W – Fear Avoidance Belief Questionnaire – Work Subscale; L- Left; N – Newtons; PPT- Pressure Pain Thresholds; R- Right; TPD - Two-point discrimination; TSK - Tampa Scale of Kinesiophobia; VAS- Visual Analogue Scale

#### Fear of movement and fear-avoidance beliefs, tactile acuity, muscle strength, and pressure pain thresholds

For fear of movement, FABQ-PA, FABQ-W, and PPT (p > 0.05) there was no significant main effect for time nor a significant interaction between time and group (p > 0.05; Table 3).

For tactile acuity, there were no statistically significant main effects for time or interactions (p > 0.05, Table 3) at any of the assessment points, except for C3-C4 where there was a time vs. group interaction (F [1, 53] = 6.27; p = 0.02; η2p = 0.10).

For muscle strength, there was a significant interaction for the flexors muscle strength (F [1, 53] = 4.83; p = 0.03; η2p = 0.08), but not for extensors or right and left side flexors (p > 0.05; Table 3).

## Discussion

This study assessed the immediate effects of observing neck movements (AO) on NP intensity, fear of movement, fear-avoidance beliefs, neck muscles’ strength, and tactile acuity in individuals with chronic idiopathic NP when compared to the effects of observing a natural environment.

Contrary to our hypothesis that AO would have a larger impact on pain intensity, a similar decrease in pain was found for both groups suggesting that both AO and observing a natural landscape have hypoalgesic effects. The control group reported a mean decrease in pain intensity of 37% and the AO group of 30%, from baseline to post-intervention, suggesting that these changes are clinically relevant [19]. In both groups, participants were asked to focus on the video and observe it with attention. It is known that attention is a cognitive mechanism that can either amplify or reduce pain awareness [44]. Another mechanism that might explain the reduction in pain intensity, particularly in the control group, is distraction-induced analgesia. Neuroimaging studies have shown that distraction activates the endogenous pain-inhibitory system from areas such as the prefrontal cortex, the perigenual anterior cingulate cortex, and the periaqueductal gray, as well as the posterior thalamus [45–47], while several areas of the pain matrix showed reduced activation [45]. Thus, attention and distraction from pain might have contributed to hypoalgesia in both groups. Also, it has been suggested that visualizing images depicting nature may induce lower sensory pain responses [48] as well as a relaxation response [49], further contributing to hypoalgesia in the control group. In the AO group, participants were also asked to imagine the execution of the movements they were observing and the observation of movements has been shown to induce changes in cortical activity, including increased motor cortical activity as well as excitability, which can modulate the pain networks [50, 51] and contribute to hypoalgesia. Moreover, the individuals performing the exercises in the video had a neutral facial expression, not depicting any difficulty or pain while executing the movements [16]. This could have facilitated the dissociation between pain and movement. Nevertheless, the decrease in pain intensity and the mental practice of the exercises did not result in increased strength in the AO group. Potentially, this might be due to the unfamiliarity of participants with the exercises. A previous study has suggested that observing a familiar action activates the sensorimotor maps relative to the person’s experience, but if the viewer observes unfamiliar movements, the motor system could in turn be inhibited [52]. These might suggest that the choice of movement for mental practice might have an impact on the results. In addition, the lack of similarity between the observed movements and the movements and positions used to assess neck muscle strength might also have contributed to the absence of impact. It has been suggested that observation of an action increases motor excitability specific to the muscles involved in the observed action [54]. Future studies should explore whether familiarity and previous execution of the imagined movement impact the results of AO.

Previous studies using a single session of AO in patients with NP have reported increased PPT in one body site out of three measured in the neck [55], and increased PPT in the neck immediately after the intervention, but not at 10 min after the intervention [10]. The PPT measurements in our study were taken more than 10 min after the intervention due to the large number of measurement outcomes that were assessed. Taken together, the results might suggest that an eventual hypoalgesic effect resulting from a single session of AO is of extremely short duration.

We were unable to find any study on individuals with NP that investigated the impact of AO on psychological variables, two-point discrimination, or strength. Our finding of a significant interaction between time and group favoring the control group for two-point discrimination at C3-C4 was not expected. A study demonstrated through magnetic resonance imaging that observing video clips of a touched hand activates the somatosensory cortices due to the mirror neuron system [56]. However, the difference was found only for one measurement site out of six. We found that assessing tactile acuity requires a lot of attention on the side of the participant which caused fatigue and impatience and might have impacted results. Future studies should use shorter protocols of assessment and verify the current study results.

We found no changes in kinesiophobia or fear-avoidance beliefs both in the AO group and in the control group, despite the decrease in pain intensity. It has been suggested that observing movements perceived as potentially harmful or dangerous activates the sympathetic nervous system, due to fear [53]. Participants in the AO group might have perceived the movements as being harmful. Similarly to our findings, a previous study [57] showed that AO in addition to exercises does not impact kinesiophobia in comparison with exercise only, in patients with chronic pain. Future qualitative research is of great relevance to help understand the cognitions, feelings, and perceptions of patients with pain towards AO and whether this can impact AO’s potential effects.

### Clinical implications and future research

The results of this study suggest that both observing neck exercises and natural landscapes may be used in clinical practice to reduce pain, potentially facilitating the administration of exercise and complementing pain education. Participants’ perceptions of the intervention were not assessed, but a few participants reported feeling relaxed after watching the natural landscape video and a decrease in the muscle tension in the neck region after the AO. These variables can be assessed in future studies. Future studies should investigate the effects of administering AO for longer periods and different dosages.

### Limitations of the study

The present study results need to be interpreted considering its limitations. Assessors were not blind to the intervention received by each participant, which could have increased type I errors. Only immediate effects were assessed. The lack of a previously established and validated protocol of AO therapy in the treatment of chronic pain places a critical question as to whether the protocol followed in this study had the appropriate duration. The participants’ attention or the ability to imagine the movement being observed were not assessed and these might be critical for the success of AO. Nevertheless, an effort was made to minimize distraction when participants were receiving the intervention by administering it in a quiet room with white walls and nothing more than the table where the computer was placed and the chair where the participant sat.

## Conclusion

Results suggest a similar acute benefit for both a single session of AO and observing natural landscapes for promoting hypoalgesia, but no impact on kinesiophobia, fear-avoidance beliefs, or PPTs. Also, AO had no positive effect on two-point discrimination and muscle strength. Further research is needed, with longer interventions and a qualitative component that considers patients’ perceptions towards the intervention.

## Data Availability

The datasets used and/or analysed during the current study are available from the corresponding author upon reasonable request.

## Abbreviations

AO: Action Observation
Cm: Centimeters
indFABQ-PA: Fear Avoidance Belief Questionnaire – Physical Activity Subscale
FABQ-W: Fear Avoidance Belief Questionnaire – Work Subscale
Kg: Kilograms
L: Left
NDI: Neck Disability Index
NP: Neck Pain
N: Newtons
PCS: Pain Catastrophizing Scale
PPT: Pressure Pain Thresholds
R: Right
SD: Standard Deviation
TPD: Two-point discrimination
TSK: Tampa Scale of Kinesiophobia
VAS: Visual Analogue Scale
